# Exploring a prolonged enterovirus C104 infection in a severely ill patient using nanopore sequencing

**DOI:** 10.1093/ve/veab109

**Published:** 2022-03-18

**Authors:** Hayley Cassidy, Leonard Schuele, Erley Lizarazo-Forero, Natacha Couto, John W A Rossen, Alex W Friedrich, Coretta van Leer-Buter, Hubert G M Niesters

**Affiliations:** Department of Medical Microbiology and Infection Prevention, University of Groningen, University Medical Center Groningen, Hanzeplein 1, Groningen 9713 GZ, The Netherlands; Institute of Medical Microbiology and Hygiene, University of Tübingen, Elfriede-Aulhorn-Str. 6, Tübingen 72076, Germany; Department of Medical Microbiology and Infection Prevention, University of Groningen, University Medical Center Groningen, Hanzeplein 1, Groningen 9713 GZ, The Netherlands; Department of Medical Microbiology and Infection Prevention, University of Groningen, University Medical Center Groningen, Hanzeplein 1, Groningen 9713 GZ, The Netherlands; Milner Centre for Evolution, Department of Biology and Biochemistry, University of Bath, Claverton Down, Bath BA2 7AY, UK; Department of Medical Microbiology and Infection Prevention, University of Groningen, University Medical Center Groningen, Hanzeplein 1, Groningen 9713 GZ, The Netherlands; Department of Pathology, University of Utah School of Medicine, 15 North Medical Drive East, Salt Lake City, UT 84112, USA; Department of Medical Microbiology and Infection Prevention, University of Groningen, University Medical Center Groningen, Hanzeplein 1, Groningen 9713 GZ, The Netherlands; Department of Medical Microbiology and Infection Prevention, University of Groningen, University Medical Center Groningen, Hanzeplein 1, Groningen 9713 GZ, The Netherlands; Department of Medical Microbiology and Infection Prevention, University of Groningen, University Medical Center Groningen, Hanzeplein 1, Groningen 9713 GZ, The Netherlands

**Keywords:** enterovirus, chronic infection, virus evolution, immunocompromised, nanopore sequencing, intra-host evolution

## Abstract

Chronic enterovirus infections can cause significant morbidity, particularly in immunocompromised patients. This study describes a fatal case associated with a chronic untypeable enterovirus infection in an immunocompromised patient admitted to a Dutch university hospital over nine months. We aimed to identify the enterovirus genotype responsible for the infection and to determine potential evolutionary changes. Long-read sequencing was performed using viral targeted sequence capture on four respiratory and one faecal sample. Phylogenetic analysis was performed using a maximum likelihood method, along with a root-to-tip regression and time-scaled phylogenetic analysis to estimate evolutionary changes between sample dates. Intra-host variant detection, using a Fixed Ploidy algorithm, and selection pressure, using a Fixed Effect Likelihood and a Mixed Effects Model of Evolution, were also used to explore the patient samples. Near-complete genomes of enterovirus C104 (EV-C104) were recovered in all respiratory samples but not in the faecal sample. The recovered genomes clustered with a recently reported EV-C104 from Belgium in August 2018. Phylodynamic analysis including ten available EV-C104 genomes, along with the patient sequences, estimated the most recent common ancestor to occur in the middle of 2005 with an overall estimated evolution rate of 2.97 × 10^−3^ substitutions per year. Although positive selection pressure was identified in the EV-C104 reference sequences, the genomes recovered from the patient samples alone showed an overall negative selection pressure in multiple codon sites along the genome. A chronic infection resulting in respiratory failure from a relatively rare enterovirus was observed in a transplant recipient. We observed an increase in single-nucleotide variations between sample dates from a rapidly declining patient, suggesting mutations are weakly deleterious and have not been purged during selection. This is further supported by the persistence of EV-C104 in the patient, despite the clearance of other viral infections. Next-generation sequencing with viral enrichment could be used to detect and characterise challenging samples when conventional workflows are insufficient.

## 1. Introduction

Chronic infections in immunocompromised patients can cause a significant burden in intensive care units (ICUs) within hospitals. Patients receiving immunosuppressive drugs, due to transplantation or undergoing chemotherapy, have an increased risk of developing infections, which may become chronic without appropriate immune system activation. Although enteroviruses are highly prevalent within the community, they are usually self-limiting and resolved within a few weeks (Genoni et al. 2017). The majority of individuals present with mild symptoms, ranging from gastroenteritis to respiratory distress, especially in children. However, enterovirus infections can be particularly problematic in patients with a weakened immune system. Indeed, studies have shown that some enteroviruses can persist for years in the body and can be continually shed in faeces (Laassri et al. 2018).

Enterovirus C104 (EV-C104) is a genotype within group C and has been associated with respiratory presentation and disease (van Leer-Buter et al. 2016). Since its first identification from a sample collected in Switzerland in 2005, it has been reported sporadically worldwide, including in Italy and Gambia in 2013, Japan in 2012, and Belgium in 2020 (Kaida et al. 2012; Piralla et al. 2013; Tokarz et al. 2013; Wollants et al. 2020). Other clinical presentations of EV-C104 have been reported previously, including seizures in a patient with leukaemia from the Netherlands in 2014 (van Leer-Buter et al. 2016). Little is known about the persistence of EV-C104 in patients. However, poliovirus, one of the most well-studied group C enteroviruses, has been known to persist in the body for over 30 years (Laassri et al. 2018).

A rapid diagnosis of enterovirus infections is important to facilitate infection control and rule out other potential pathogens to prevent unnecessary empiric antibiotic treatment. Enteroviruses are ss-RNA viruses with approximately 7,500 nucleotides in length and are members of the *Picornaviridae* family (Joffret et al. 2018). Real-time reverse-transcriptase polymerase chain reaction (RT-qPCR) is used for enterovirus detection, while Sanger sequencing remains the gold standard in routine molecular virology for genotype characterisation by targeting the viral protein 1 (VP1) or VP4 gene encoding the viral capsid (Harvala et al. 2018). Enteroviruses are known to have vast variations in their genomes (Simmonds et al. 2006) and are prone to recombination events, which have been suggested to be a driving force for evolution (Song et al. 2020). Untypeable samples can be increasingly troubling, particularly when linked to unique or severe clinical presentation. Next-generation sequencing (NGS) technology has been used previously to determine and characterise challenging patient samples (Greninger 2018; Schuele et al. 2020). By obtaining complete or near-complete genomes, more information can be gathered on evolution patterns and variations in primer binding sites to refine targeted conventional assays, such as routine RT-qPCR and Sanger sequencing.

Here, we describe a fatal case of a patient with a chronic untypeable (through Sanger sequencing) enterovirus infection over nine months. Viral targeted sequence capture was used in combination with Oxford Nanopore Technologies (ONT) sequencing to determine the enterovirus genotype responsible for the infection, to potentially link this genotype to disease progression and to reveal evolutionary changes.

### 1.1 Patient clinical timeline

The case describes a 69-year-old immunocompromised patient with atrial fibrillation and autologous stem cell transplantation (2009), following a multiple myeloma stage 2 (IgG-k) diagnosis in 2008. The patient initially received thalidomide and velcade with dexamethasone treatment (VelDx) before achieving remission in 2013 and subsequent treatment with lenalidomide with dexamethasone (LenDex). After a multiple myeloma relapse in 2015, the patient underwent an extensive seventh line therapy with daratumumab and dexamethasone until his death in 2019. In November 2018, the patient developed a fever along with a cough and was admitted for five days due to a suspected respiratory infection. A screen on the BioFire FilmArray v2.0 respiratory panel identified an enterovirus and human parainfluenza virus type 4 (HPIV-4) in the nasopharyngeal swab sample. Two weeks later, in December 2018, the patient was admitted again, this time for six days after presenting with dyspnoea and rectal bleeding. Only an enterovirus was identified in the nasopharyngeal swab sample. At the beginning of January 2019, the patient was admitted for another seven days for a suspected pneumococcal infection. Three weeks later, the patient was observed at the emergency department for another episode of dyspnoea from a suspected respiratory infection. A respiratory screen on the BioFire FilmArray identified an enterovirus and coronavirus OC43 (CoV-OC43) in the nasopharyngeal swab sample.

Finally, in June 2019, the patient was diagnosed with a bilateral pneumonia after presenting with fever and dyspnoea in the emergency department. Only an enterovirus was identified at this time in the nasopharyngeal swab sample. The patient initially received piperacillin and tazobactam, followed by ciprofloxacin (which are routinely administered); however, respiratory insufficiency continued to increase. Following seven days in the ICU, the patient unfortunately died. From November 2018 to June 2019, the patient had a positive enterovirus detection following each hospital admission. Five samples (collected over nine months) were selected for further analysis. Table 1 and S1 provide detailed clinical information, beginning with the first enterovirus detection and hospital admission.

**Table 1.** Clinical timeline.

Sample ID	Date of collection	Sample type	RT-qPCR Ct	Clinical presentation	Length of stay/department	Co-detection	Diagnosis on discharge^a^	Outcome of disease episode
Respiratory 1	27 November 2018	NS	23	Fever, malaise, and productive cough	5 days (internal medicine)	HPIV-4	Respiratory infection	Home, but remains very tired from respiratory infection
Respiratory 2	17 December 2018	NS	28	Shortness of breath	6 days (internal medicine)	None	Anaemia and heart failure	Home (no more shortness of breath)
Respiratory 3	28 January 2019	NS	18	Shortness of breath, thoracic pain, productive cough, and diarrhoea	1 day (emergency department)	CoV-OC43	Viral respiratory infection	Still weak. No more cough and fever
Respiratory 4	30 June 2019	NS	25	Shortness of breath, cough, and low oxygen saturation	7 days (ICU)	None	Enterovirus bilateral pneumonia. Renal dysfunction led to hypotension	No recovery, continued hospital stay
Faecal 1	02 July 2019	Faeces^b^	28	Continued shortness of breath, fever, and respiratory failure	7 days (ICU)	None	Enterovirus bilateral pneumonia	No recovery, increasing dyspnoea. Mortality

**Abbreviations:** Ct, cycle threshold; NS, nasopharyngeal swab; CoV-OC43, coronavirus OC43; ICU, intensive care unit. ^a^Final diagnosis on discharge, according to the attending clinician. ^b^Only a faecal sample was collected during this time period.

## 2. Materials and methods

### 2.1 Ethics statement

Oral consent for the use of clinical samples for research purposes is routinely obtained upon patient admission to the University Medical Center Groningen (UMCG). This study was evaluated by the local UMCG Ethics Committee and a waiver was obtained, METc-2021/284. All experiments were performed in accordance with the guidelines of the Declaration of Helsinki and the institutional regulations. All samples and patient data were anonymised according to local guidelines.

### 2.2 Sample selection

A total of five samples (four respiratory and one faecal) were included in the study with a positive enterovirus detection (Table 1). Each sample was collected from the patient after respiratory symptoms were observed during hospital admission. The respiratory samples were initially tested on the FilmArray BioFire v2.0 respiratory panel (BioMérieux, Salt Lake City, Utah, United States of America), which includes seventeen viral targets and three bacterial targets. An RT-qPCR was then performed to distinguish between rhinovirus and enterovirus results generated from the FilmArray (Poelman et al. 2015). A gastrointestinal screen was then performed on the faecal sample using a further RT-qPCR. Sanger sequencing was subsequently performed on samples with a high viral load (Ct <30) by targeting the VP1 gene on the viral capsid to determine genotype characterisation (Nix et al. 2006). However, no genotype could be determined.

### 2.3 ONT sequencing

To obtain the complete/near-complete enterovirus sequences, a combination of viral enrichment using the ViroCap share developer panel (Wylie et al. 2015) SeqCapEZ HyperCap 2.1 from Roche NimbleGen (Madison, WI, USA) and long-read sequencing was performed. Briefly, samples were first centrifuged at 6,000 × *g* for 2 min. Total nucleic acids were extracted from 190 µL of the supernatant using the easyMAG (BioMérieux, Inc., Marcy l’Etoile, France). Nucleic acids were concentrated to 8 µL using the RNA clean and concentrator-5 kit (Zymo Research, Irvine, USA), including an in-column DNase treatment using TurboDNase I (ThermoFisher Scientific, Waltham, USA), according to the manufacturer’s instructions. Complementary DNA (cDNA) was synthesised using a Sequence-Independent Single-Primer-Amplification (SISPA) approach using round A/B primers (5′-GTTTCCCACTGGAGGATA-N9-3′; 5′-GTTTCCCACTGGAGGATA-3′) as described previously (Greninger et al. 2015). cDNA was subsequently enriched using the ViroCap share developer panel (NimbleGen, Madison, USA) following the modifications by Schuele and colleagues for ONT sequencing (Schuele et al. 2020). Sequencing libraries were generated from 250 ng of enriched cDNA using the Ligation Sequencing Kit (SQK-LSK109) (ONT, Oxford, UK) and native barcoding expansion kit (EXP-NBD104) (ONT), according to the manufacturer’s instructions. Barcoded libraries were pooled together by equal mass and sequenced on an FLO-MIN106 R9.4.1 flowcell (ONT) using a MinION device (ONT).

### 2.4 Data analysis

Raw reads were first basecalled with a high accuracy mode, trimmed and demultiplexed with the double barcode option using Guppy (v4.0.9) (ONT) (Table S2). Reads were subsequently further trimmed by Porechop (v0.2.3) (https://github.com/rrwick/Porechop) (Table S2). CLC Genomics Workbench (v20.0.3) (CLC) (Qiagen, Aarhus, Denmark) was used to trim SISPA A/B primer sequences and to remove human reads (hg19). Next, Filtlong (v0.2.0) (https://github.com/rrwick/Filtlong) was applied to remove 10 per cent of the lowest quality reads and sequences <300nt (Table S2). Trimmed reads were uploaded onto Genome Detective (v1.132) (Vilsker et al. 2019), a web-based metagenomic tool to rapidly characterise enteroviruses in each sample and to determine co-detections.

CLC was used to map the trimmed reads against an in-house enterovirus database (*n* = 2,237 sequences, 6,000–8,000 nt, from 1990 to 2019 and accessed 07 March 2019) from NCBI GenBank to determine the predominant genotype. A consensus sequence was generated from the read mappings (Table S3) and cross-referenced with NCBI BLAST. The consensus was then polished 3x using Racon (v1.4.13) (https://github.com/isovic/racon) (Table S2) and Medaka (v1.1.0) (ONT) (Table S2) prior to adding annotations using the Genome Finishing Module (CLC), to illustrate sequencing depth and potential genome coverage bias. The accession numbers for the four complete/near-complete genomes generated can be found in Table 2.

**Table 2.** Enterovirus characterisation.

Sample ID	Number of total reads	Number of EV-C104 reads	Average sequence length	Average sequencing depth	Consensus length (nt)	Regions >30x^a^	EV-C104 coverage^b^ (%)	Accession number
R1	555,998	5,761	1,000	780x	7,390	194–7,208	100	MZ092702
R2	559,943	54,050	735	5,507x	7,213	22–7,146	98.4	MZ092703
R3	677,389	114,059	977	15,037x	7,411	15–7,411	100	MZ092704
R4	221,468	57,725	844	6,784x	7,154	167–7,121	97.9	MZ092705
F1	629,278	0	N/A	N/A	N/A	N/A	N/A	N/A

a Coverage depth parameter based on Oude Munnink et al. (2020) and Karst et al. (2021). ^b^MN481403.1 was initially used as a best hit reference genome; followed by KC785524.1 (7332bp) for annotation, being the closest annotated reference. Abbreviations: R1; Respiratory 1, R2; Respiratory 2, R3; Respiratory 3, R4; Respiratory 4.

To determine the phylogenetic relationships between the patient samples and the selected ten near-full (>95 per cent EV genome) reference genomes on NCBI, consensus sequences were aligned with MAFFT (v7.471). EV-C104 reference genomes were selected based on ≥99 per cent query cover and >95 per cent percentage identity. Three additional group C enteroviruses (EV-C109, EV-C105 and EV-C117) served as outgroups. The patient samples were manually edited to remove obvious sequencing errors in homopolymeric regions. This was similarly performed previously (Duchene et al. 2020). A maximum likelihood phylogenetic tree was generated using the GTR + G + I model implemented in CLC with the robustness of 1,000 bootstrap replications (Table S4). FigTree (v1.4.4) and iTOL (v6) were subsequently used for visualisation (Rambaut 2014; Letunic and Bork 2021). Next, we explored the temporal signal of our EV-C104 dataset using TempEST (v2.7.0) (Rambaut et al. 2016), which was designed to explore heterochronous data and determine the best fitting root. A maximum likelihood phylogenetic tree was similarly generated using the GTR + G + I model implemented in CLC with the robustness of 1,000 bootstrap replications without the outgroups. We subsequently performed a time-scaled phylogenetic analysis using TimeTree v0.8.1 (Sagulenko et al. 2018) and the augur pipeline implemented in Nextstrain (Hadfield et al. 2018). Phylodynamic analysis was performed on the maximum likelihood tree using a strict molecular clock, a coalescent Skyline tree prior and the best root fitted. The time-scaled tree was then visualised using auspice (v0.8.0) (Hadfield et al. 2018).

Expected primer annealing sites from the Sanger sequencing targeting VP1 (325 bp) were evaluated using the ‘Find Primer Binding Sites and Create Fragments’ tool on CLC (Table S5). To determine the presence of intra-host single-nucleotide variant (SNVs), the variant finding tool on CLC was applied using the Fixed Ploidy algorithm with 80 per cent variant probability and 75 per cent minimum frequency (Table S6). Additionally, to determine positive or negative selection pressure, the MAFFT alignment (CDS regions only) was uploaded onto Datamonkey (v1.6.0) (Weaver et al. 2018), and a Fixed Effect Likelihood (FFL) model (Kosakovsky Pond et al. 2005) was performed on the patient sequences (host level), while a Mixed Effects Model of Evolution (MEME) (Murrell et al. 2012) was performed on the patient and reference EV-C104 sequences (population level) (*n* = 10) (posterior probability [PP] value = 0.1).

## 3. Results

### 3.1 Enterovirus characterisation

Genomes recovered from long-read sequencing on the MinION were subsequently identified as EV-C104 following confirmation using Genome Detective and NCBI BLASTn. The EV-C104 genotype was retrieved in respiratory samples 1–4 but not in the faecal material (Table 2). Our results suggest that EV-C104 was responsible for the prolonged respiratory infection. Four complete or near-complete EV-C104 genomes were obtained with an average sequencing depth of 7,027 × (780–15,037) and an average read length of 889 nt (735–1,001 nt) per sample (Table 2). An NCBI BLASTn search using the obtained patient genome sequences revealed a 97.63 per cent to 98.08 per cent identity to an EV-C104 sequence from Belgium (MN481403.1) collected in 2018. Overall, four co-detections were identified: HPIV-4 (respiratory sample 1), torque teno virus (TTV) (respiratory sample 2), CoV-OC43 (respiratory sample 3) and human picobirnavirus (which consisted of 41.5 per cent of reads in faecal sample 1), using ONT sequencing (Table 3). Two of these detections, CoV-OC43 and HPIV-4 had also been identified through the BioFire FilmArray in routine diagnostics (Table 1).

**Table 3.** Co-detections.

	Significant co-detections				
Sample ID	Microorganism identified	Number of reads	Consensus length	% of genome recovered	Average sequencing depth
Respiratory 1	HPIV-4	15	2,418	14.3^a^	0.7x
Respiratory 2	TTV	75	498	17.9^b^	12.4x
Respiratory 3	CoV-OC43	411,196	30,736	100^c^	12,311x
Respiratory 4	None detected	N/A	N/A	N/A	N/A
Faecal 1	Human picobirnavirus	261,110	1,717	100^d^	118,511x

**Abbreviations:** CoV, coronavirus; HPIV, human parainfluenza virus. ^a^Reference (MT118684) 16,896bp. ^b^Reference (FJ392116) 2,782bp. ^c^Reference (MT118678) 30,737bp. ^d^Reference (KJ663816.1) 1,717bp. Trimmed reads were uploaded onto Genome Detective and then mapped against the best hit reference from NCBI using CLC to determine the number of reads, average sequencing depth, and consensus length.

### 3.2 Genome coverage and primer binding

**Figure 1.**
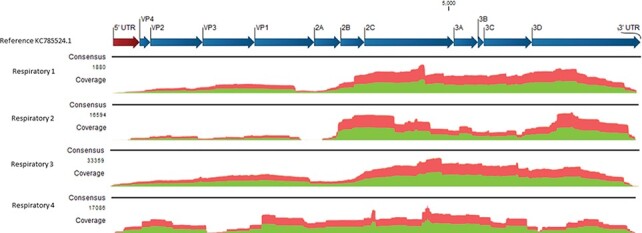
EV-C104 genome coverage. Coverage depth across EV-C104 genomes. The *y*-axis depicts the number of reads and the *x*-axis depicts the genome position. Red, single read in the reverse direction; green, single read in the forward direction. The open reading frame is depicted with blue arrows. Untranslated regions are depicted with red arrows.

A similar genome coverage pattern was achieved for each patient sample, with a higher coverage of conserved genes 2C-3D and a lower coverage of genes VP4-VP1 (Fig. 1). To investigate the effect of viral enrichment on coverage bias, the sample with the highest coverage, Respiratory 3, was also sequenced without viral enrichment (Figure S1). Similar coverage patterns were achieved, with and without enrichment indicating no coverage bias after target sequence capture. As the EV-C104 sequences were previously untypeable using Sanger sequencing, primer binding sites and potential mismatches were investigated *in silico*. Despite the presence of degenerate primers to account for enterovirus variation, mismatches were identified in the nested PCR primers used prior to Sanger sequencing (Table S7). *In silico* analysis of primer binding sites with closely related EV-C104 reference sequences (MN481403.1 and KR815824.1) also resulted in a similar mismatch result, indicating that alternative gene targets or primers may be more suitable for future EV-C104 typing using Sanger sequencing.

### 3.3 Phylogenetic analysis

**Figure 2.**
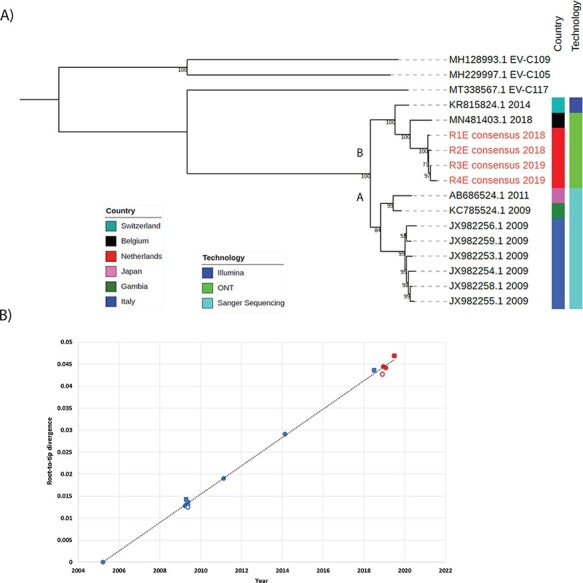
Phylogenetic reconstruction and genetic divergence of EV-C104. (A) Maximum likelihood phylogenetic tree inferred from EV-C104 complete/near-complete sequences. EV-C104 reference genomes were selected with ≥99 per cent query cover and >95 per cent percentage identity. Patient samples (in red with the letter E to indicate they have been enriched), ten complete EV-C104 genomes and three EV-C genomes from GenBank served as outgroups. CLC was used to generate the alignment and construct the tree. A General Time Reversible substitution model and a gamma distribution with invariant sites with 1,000 bootstraps were used. The emergence of two distinct genogroups A and B can be observed. Bootstrap values are shown at the branch nodes. (B) A maximum likelihood phylogenetic tree with a General Time Reversible substitution model and a gamma distribution with invariant sites with 1,000 bootstraps were generated without the outgroups. A root-to-tip regression with the *y*-axis corresponding to branch distances of the phylogenetic tree (in units of substitutions per site) and the *x*-axis corresponding to time (year). Patient sequences are in red (*n* = 4) and reference sequences (*n* = 10) are in blue. Points below the regression line indicate sequences that are less divergent than average (for their sampling date) and vice versa. Squares indicate sequences above the regression line and have more divergence; empty circles indicate sequences below the regression line and have less divergence. **Abbreviations:** Respiratory 1; R1, Respiratory 2; R2, Respiratory 3; R3, Respiratory 4; R4.

A maximum likelihood tree based on our four EV-C104 consensus sequences (Respiratory 1–4), along with ten complete or near-complete EV-C104 references from NCBI GenBank, was inferred (Fig. 2A). To create a rooted tree, three additional group C enteroviruses were used as outgroups. We found our four patient samples (shown in red) to cluster with the most recent EV-C104 sequence (MN481403.1) from Belgium in 2018. Additionally, temporal aggregation was observed within the patient samples, with increasing substitutions per site, beginning with the first sample collected in November 2018 (Figure S2). As yet, EV-C104 does not have specific subgroups; however, there appear to be two different monophyletic populations (which we have named genogroups A and B), one containing sequences from Switzerland (KR815824.1) and Belgium (MN481403.1), along with our patient sequences and another population, containing six sequences from Italy (JX982254.1–JX982259.1), along with sequences from Japan (AB686524.1) and Gambia (KC785524.1). Conversely, the sequences from the former population are all from immunocompromised patients (Table 4).

**Table 4.** Overview of complete EV-C104 genomes (*n* = 10) from GenBank.

				Patient(s)			
Accession number	Country of origin	Collection year	Technology	Age	Immune status	Clinical presentation	Reference
KC785524.1	Gambia	2009	Sanger dideoxy sequencing	Child	Unknown	Pneumonia	Tokarz et al. 2013
JX982254.1–JX982259.1 (*n* = 6)	Italy	2009	Sanger dideoxy sequencing	Adults and children	Immunocompromised and immunocompetent	Mild respiratory syndrome	Piralla et al. 2013 and Piralla et al. 2012
AB686524.1	Japan	2011	Sanger dideoxy sequencing	Adult	Immunocompetent	Respiratory viral infection	Kaida et al. 2012
KR815824.1	Switzerland	2014	Illumina^a^	Adult	Immunocompromised	Respiratory viral infection	Lewandowska et al. 2015
MN481403.1	Belgium	2018	ONT	Child	Immunocompromised	Recurrent upper respiratory infections	Wollants et al. 2020

a Illumina MiSeq for 1 × 150 cycles.

To estimate the evolutionary changes of EV-C104 between the sample dates, branch lengths from an additional maximum likelihood tree without the outgroups were used to generate a root-to-tip-regression analysis (Fig. 2B). The EV-C104 phylogeny exhibits a strong association between genetic distances and sampling dates (*R*^2^ = 0.998). Moreover, the regression analysis confirmed heterogeneity among the EV-C104 sequences with an estimated recent common ancestor in 2005 (middle of 2005) (Fig. 2B). Meanwhile, the patient EV-C104 sequences alone were shown to have an estimated recent common ancestor in the middle of 2018. A time-scaled phylogenetic analysis estimated an evolution rate of 2.97 × 10^−3^ substitutions per year. The time-scaled phylogenetic tree is visualised in Figure S3. Furthermore, three EV-C104 sequences can be observed above the regression line: JX982254.1 (Italy), MN481403.1 (Belgium), and Respiratory 4, indicating a higher divergence. Interestingly, the EV-C104 from Belgium was also associated with a recurrent respiratory infection (Table 4). Meanwhile, two EV-C104 sequences can be seen below the regression line, JX98258.1 (Italy) and Respiratory 1, indicating a lower divergence.

### 3.4 Viral evolution

**Figure 3.**
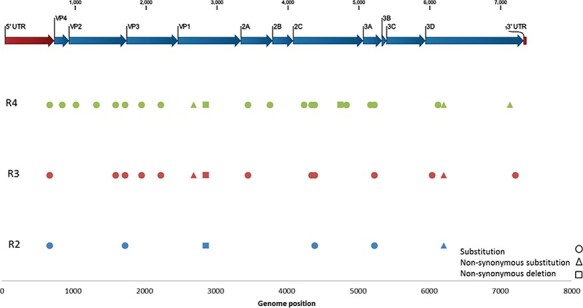
Intra-host variants between patient samples. A Fixed Ploidy algorithm was used with 80 per cent variant probability and 75 per cent minimum frequency. To determine variation between sampling dates, Respiratory 2–4 were compared against Respiratory 1.

**Abbreviations:** Respiratory 2; R2, Respiratory 3; R3, Respiratory 4; R4.

As the patient was shown to have an apparent chronic EV-C104 infection, it could be plausible that the virus may have a different evolutionary pattern, compared to an immunocompetent patient with an intact immune system. As a result, we investigated single-nucleotide variants (SNVs) in the genome of the four patient samples, from the first sample collected in November 2018 to the last sample collected in June 2019, to determine intra-host variation (Fig. 3 and Table S8). SNVs were observed throughout the genome, in both structural and nonstructural segments of the polyprotein, with an increasing frequency over time (3.7-fold change from Respiratory 2 to Respiratory 4) (Fig. 3). Genome changes occurred predominantly in the 2C, VP3, and 3D genes, which make up approximately 3.6 per cent, 9.8 per cent, and 18.8 per cent of the viral genome, respectively, but harboured 19 per cent (*n* = 8), 16.7 per cent (*n* = 7), and 16.7 per cent (*n* = 7) of the observed changes. A total of ten nonsynonymous mutations were identified (Fig. 3 and Table S8), most commonly in the VP1 gene (the receptor-binding domain). Interestingly, two of these nonsynonymous mutations at genome positions 2,863 and 6,204 were retained from Respiratory 2 to Respiratory 4 (Fig. 3). Firstly, a nucleotide (G2863) deletion in the VP1 gene led to a change in amino acid Asp723 and a frameshift mutation with a 75.7–78.7 per cent frequency. Secondly, a nucleotide substitution (T6204A) in the 3D gene led to a change in amino acid Asp1834Glu with a 93–94.2 per cent frequency.

**Figure 4.**
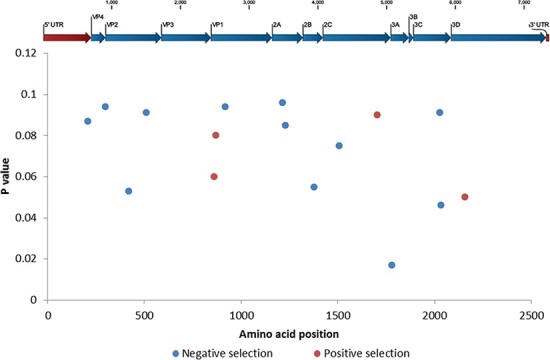
Positive and negative selection pressure on individual codon sites. Only the patient genomes were used to analyse negative purifying selection (PP value 0.1). All EV-C104 genomes (*n* = 14) used in the regression analysis in Fig. 2b were used to analyse positive diversifying selection (PP value 0.1).

An FFL model was used to infer the type of selection acting on the nonsynonymous and synonymous substitutions for each codon site in the EV-C104 genomes and therefore more suited for studying samples from the same patient. Negative selection was subsequently found at twelve sites (with the PP value set at 0.1—occurring 90 per cent of the time) scattered throughout the genome (Fig. 4), including sites involved in the viral capsid formation, viral replication, and maturation. An MEME model was used to determine the type of selection occurring at a population level (patient samples and references) by measuring the distribution of variation, not only from site to site (similarly to the FFL model) but also from branch to branch. Interestingly, an MEME model found evidence of an episodic positive or diversifying selection at four codon sites (PP value 0.1) using all EV-C104 sequences in the regression analysis in Fig. 2B, with two out of the four sites from the VP1 gene and the other two in the 2C and 3D genes (Fig. 4).

## 4. Discussion

Here we describe an immunocompromised patient with a chronic enterovirus infection contributing to a fatal bilateral pneumonia. We applied viral targeted sequence capture and ONT long-read sequencing to determine EV-C104 as the responsible genotype for disease progression and to follow its evolution in an immunocompromised patient. To the best of our knowledge, this is the first study to report a persistent EV-C104 infection in a patient and the first complete EV-C104 genome from the Netherlands.

Since its first description in Switzerland in 2009 (Tapparel et al. 2009), EV-C104 has been reported sporadically worldwide (Wollants et al. 2020). Currently, NCBI GenBank has only thirteen complete and twenty-one partial EV-C104 genomes. This study generated an additional four complete/near-complete EV-C104 genomes (GenBank accession numbers: MZ092702–MZ092705). EV-C104 has been associated with respiratory disease both in children and in adults (Kaida et al. 2012; Tokarz et al. 2013; Piralla et al. 2013; Lewandowska et al. 2015; Wollants et al. 2020). However, it has also been speculated to infect the central nervous system (Tapparel et al. 2009). From the limited studies available, it appears that an advanced age and a compromised immune status may be a prerequisite for severe disease (Table 4).

NGS approaches can characterise and detect all viruses directly from clinical samples (Lewandowski et al. 2019) and have been applied previously to type enteroviruses (Wollants et al. 2020; Schuele et al. 2020). ONT sequencing platforms enable long-read sequencing, real-time analysis, and lower starting prices, opening up sequencing to a broader scientific community (McNaughton et al. 2019). However, ONT sequencing still generates a lower output and a higher error rate compared to Illumina sequencing (Lewandowski et al. 2019). As a result, ONT reads and consensus sequences require further downstream processing to increase accuracy. Enrichment strategies are often needed to increase the sensitivity of NGS approaches (Lewandowski et al. 2019; Fitzpatrick et al. 2021). We applied viral enrichment using a broad panel of oligonucleotide capture probes to increase sensitivity, while retaining the breadth of metagenomic approaches (Briese et al. 2015). This enabled us to obtain high-quality consensus sequences with sufficient coverage depth for typing, SNV calling, and evolutionary analyses from a range of Ct values. Despite obtaining four complete/near-complete genomes, we could not obtain a single enterovirus read from the faecal sample (Table 1). Interestingly, EV-C104 has not yet been reported in faecal samples previously. A possible explanation could be the over-representation of human picobirnavirus (41.5 per cent of total reads) (Greninger 2018) in the faecal sample and the high abundance of bacterial background, which may have led to fewer sequencing reads for EV-C104 (Pallen 2014).

With the method used for this report, we also detected HPIV-4 and CoV-OC43, confirming the routine diagnostic results (Tables 1 and 3). No reads could be assigned from the negative control, which was run parallel to the patient samples. In addition, we also detected the expression of TTV and the presence of human picobirnavirus. TTV and human picobirnavirus are still under consideration as etiological agents as information regarding pathogenesis and involvement in disease remains unknown. However, TTV has been recently reported as a potential endogenous marker of immune function in transplant patients (Rezahosseini et al. 2019). The finding of this virus in our case could reflect the immune-suppressed condition of this autologous stem cell recipient. Although human picobirnavirus has been associated with gastrointestinal disease (Ghosh and Malik 2021), the gastrointestinal symptoms listed during the collection of Respiratory 2 and 3 were more likely a side effect from the antibiotic and immunosuppressive treatment. Interestingly, only EV-C104 was consistently found throughout the sampling period, compared to CoV-OC43 and HPIV-4, suggesting that EV-C104 had a higher potential for causing chronic infection in this patient.

Enteroviruses have vast variations in their genomes due to frequent mutations and recombination events (Grädel et al. 2019), which may influence the significant variability in clinical presentation. Although Sanger sequencing is considered the gold standard, there has been a fluctuating success rate of enterovirus genotyping. Indeed, untypeable enteroviruses have been observed previously from 8.5 per cent to 24.8 per cent of samples (van Leer-Buter et al. 2016; Gelaw et al. 2020; Posnakoglou et al. 2021). Genotyping is essential to link clinical manifestations with specific types, track outbreaks and guide the development of new diagnostic tests or potential therapies. Mismatches occurring at the 3′ end of the primer have been known to have the most detrimental effects in detection (Stadhouders et al. 2010) and can lead to false negatives. Primer analysis (Table S7) indicated high mismatching in the 3′ end in both the forward and the reverse primers and could provide a possible explanation why the enteroviruses were unable to be genotyped using Sanger sequencing. Challenges in typing EV-C104 using VP1 primers have been observed previously due to nucleotide differences at the binding sites (Lewandowska et al. 2015). However, as only four sequences from the same patient were investigated in this study, the numbers are too low to draw definitive conclusions for our inability to acquire a sequence by Sanger sequencing in this case. Nevertheless, the addition of subsequent EV-C104 sequences in the future onto public databases could facilitate the design of more appropriate primers. As it is not feasible to perform genotype-specific assays on all enteroviruses, which are rare or highly variable, NGS can be applied to complement diagnostics.

Phylogenetic analysis of the patient samples indicated a high genetic similarity (97.63 per cent—98.08 per cent identity) to a Belgium sequence and subsequently clustered on the maximum likelihood tree (Fig. 2A). Temporal aggregation was observed between the patient samples with increasing substitutions per site over time (Figure S2). This is consistent with a persistent infection and has been observed previously in an immunocompromised patient with a chronic enterovirus infection (Laassri et al. 2018). Phylogenetic analysis revealed two distinct genogroups, referred to as A and B in Fig. 2A. Remarkably, genogroup B was all sampled from immunocompromised patients (Table 4), suggesting a potential prerequisite for this particular genogroup. However, at this time, more complete genomes and clinical information are required to draw definitive conclusions.

Time-scaled phylogenetic analysis and root-to-tip regressions can be applied to provide estimates on the evolutionary rate and divergence over time. TempEst uses distance to estimate the most recent common ancestor (MRCA) by generating a linear regression using the sample collection dates (Fig. 2B). Interestingly, the MRCA, which was estimated to occur in the middle of 2005, coincided with the first detection of EV-C104 from samples collected between 2004 and 2007 (Tapparel et al. 2009). We have reported the first substitution rate for EV-C104. Our estimated rate of 2.97 × 10^−3^ for the entire polyprotein was similar to that observed for the VP1 gene of enterovirus A71, ranging from 3.60 × 10^−3^ to 5.345 × 10^−3^ (Bessaud et al. 2014; Puenpa et al. 2021) and the VP1 gene of enterovirus D68 with 4.93 × 10^−3^ (Linsuwanon et al. 2012). Further research is required, along with additional full-length EV-C104 genomes to investigate evolutionary patterns, particularly within intra-hosts.

Previous studies have revealed that different enterovirus fragments evolve independently and can combine easily, allowing a high level of flexibility and capability for rapid evolutionary changes in response to an environmental stimulus (Lukashev et al. 2005; Muslin et al. 2019). A chronic infection provides a unique snapshot of EV-C104 evolution. We investigated SNVs (variants occurring at the read level) to determine inter-host variability and track the evolutionary pattern of EV-C104 in our patient samples (Fig. 3). An increase in SNVs over time was observed, most frequently in VP3 (nt 1,716–2,438), 2C (nt 4,065–5,051) and 3D (nt 5,931–7,313) genes. Previous studies investigating enterovirus evolution have found that changes in the genome, such as recombination, are less likely to be randomly distributed and usually occur in ‘hotspots’, which are more likely to favour viable genomes (Muslin et al. 2019). Interestingly, other studies investigating poliovirus found hotspots in the 2C and 3D genes, along with sites in the 5′UTR and between VP1-2A and 2A-2B genes (McWilliam et al. 2009; Lowry et al. 2014; Muslin et al. 2019). The 2C gene (which transcribes ATPase) and the 3D gene (which transcribes RNA-dependent RNA polymerase) are highly conserved and are heavily involved in the enterovirus life cycle (Wang et al. 2020). Unsurprisingly, all but one mutation in the 2C gene was observed in the nonfunctional regions (Wang et al. 2012), suggesting a reason why the virus was still viable. The VP3 gene, on the other hand, is highly variable due to its role in host receptor binding and immune evasion.

The majority of nonsynonymous mutations occurred in the VP1 gene, with an increasing frequency between sample dates (Fig. 3). This suggests the virus may well be adapted within the host, with mutations being weakly deleterious and not necessarily purged during selection. This may have contributed to the clinical decline, coupled with the patient’s other comorbidities. Additionally, it could reveal pressure from the patient’s immune system, however, not enough to clear the virus. Nonsynonymous mutations were also observed in the 2C and 3D genes and were similarly retained between sampling dates (Fig. 3). There appeared to be a frameshift mutation retained in the VP1 gene. Interestingly, the mutation occurred in the genome position 2,863 (codon 141 in the VP1 gene), which contains the DE loop, known to be highly diverse and has frequent mutations (Dyrdak et al. 2019). BC and DE loops in enteroviruses have been revealed to be putative epitopes for neutralising antibodies, indicating the patient’s limited immune response may still have impacted EV-C104 evolution. An additional nonsynonymous substitution was observed in Respiratory 2 to 4 in the 3D gene at genome position 6,204 (codon 91 in the 3D gene). Mutations in the 3D polymerase have been found to either negatively or positively affect recombination frequency but not necessarily affect viral fitness (Xia et al. 2015; Muslin et al. 2019). Alanine substitutions in the 3D gene have also been observed previously; however, the one observed in the patient has not yet been described (Kempf et al. 2016).

Genome changes are usually retained in the progeny if they enable better survival or discarded if they have no added benefit (Muslin et al. 2019). While Respiratory 1 appears to have less divergence, Respiratory 4 has higher variation than expected for the sampling date (Fig. 2B). Lower divergence can occur through poor sequencing quality, an error in the alignment or a phylogenetic interference (Rambaut et al. 2016). Recombination or in our case mutation could account for an increased divergence (Rambaut et al. 2016). Similarly to Respiratory 4, the Belgian sequence also had a higher-than-average divergence and was also collected from an immunocompromised patient (Fig. 2B). This suggests favourable conditions for virus evolution and reflects the increasing SNV observed over time. By the time Respiratory 4 was collected, the patient was in clinical decline and died five days later in the ICU. An inadequate immune response would allow the virus to replicate continuously at a significant level.

It could be reasoned that an immunocompromised patient may have a different kind of selection shaping the evolution of EV-C104. Interestingly, an overall negative selection was determined for the EV-C104 sequences from the patient (host level) using an FFL model (Fig. 4). This could imply that while the virus has been allowed to replicate and spread, it is not necessary retaining new mutations in the progeny. This could be due to a reduced need, owing to the patient’s limited immune system, to preserve new mutations involved in host evasion or survival. Using an MEME model, selection was also investigated for all sequences including the references used in the regression analysis (population level) and identified a positive or diversifying selection in four codon sites (Fig. 4). As this analysis now includes sequences from immunocompetent patients (Table 4), with immune systems able to limit viral survival, it could account for the retention of new mutations.

Our study does have some limitations. To obtain high-quality full-length consensus sequences for evolutionary analysis and SNV calling, time-consuming enrichment and bioinformatics procedures are required. Furthermore, despite enrichment, no enterovirus reads were obtained from the faecal sample, suggesting a detection limit. Finally, with the application of DNase treatment to increase viral sensitivity of RNA viruses, only DNA viruses that were actively transcribed during sampling, such as TTV, were detectable.

## 5. Conclusion

This study highlights the potential for a persistent or chronic EV-C104 infection in a patient with an immunocompromised status. Despite the detection of clinically relevant viruses during the course of disease (CoV-OC43 and HPIV-4), the relatively uncommon EV-C104 was the only virus persistent in the patient during the whole period. The patient’s weakened immune system most likely contributed to viral diversification, with a negative selection pressure determined as the main evolutionary driver whereby new mutations were discarded. This study shows the possibilities of applying NGS to complement conventional diagnostics by adding further resolution to identify and characterise possible etiological agents.

## Supplementary Material

veab109_Supp

## Data Availability

The EV-C104 genomes reported in this study have been deposited in the NCBI GenBank with accession numbers: MZ092702–MZ092705. The sequencing reads were deposited under the BioProject: PRJNA768768.
