# Supplementary material for: Exploring a prolonged enterovirus C104 infection in a severely ill patient using nanopore sequencing
Source: Virus Evol. 2022 Mar 18;8(1):veab109. doi: 10.1093/ve/veab109 (PMC8932292; doi:10.1093/ve/veab109)
Supplement: veab109_Supp [file veab109_supp.zip › veab109_Supp/Supplementary_VEVOLU-2021-097.R2.docx]

**Supplementary Material**

# Prolonged enterovirus C104 infection in a severely ill patient using nanopore sequencing

Hayley Cassidy*^1^, Leonard Schuele*^1,2^, Erley Lizarazo-Forero^1^, Natacha Couto^1,3^, John W.A. Rossen^1,4^, Alex W. Friedrich ^1^, Coretta van Leer-Buter^1^ and Hubert G.M. Niesters^1#^

*These authors contributed equally

^#^Corresponding author

**Table S1.** Biochemical and physical examination of the patient

| **Sample ID** | **Biochemical examination** | **Physical examination** |
| --- | --- | --- |
| Respiratory 1 | C-Reactive Protein: 124 Leucocytes: 9.7 | Temperature: 38.3 °C Oxygen saturation: 97% |
| Respiratory 2 |  | Temperature: 36.4 °C Oxygen saturation: 99% |
| Respiratory 3 |  | Oxygen saturation: 99% |
| Respiratory 4 | C-Reactive Protein: 95  Leucocytes: 2.7 | Temperature: 36.8 °C  Oxygen saturation: 100%  CT scan: bilateral infiltrative abnormalities |
| Faecal 1 | C-Reactive Protein: 260  Leucocyte: 5.0 | Temperature: 35.9 °C Oxygen saturation: 93% Chest X-ray: extensive bilateral infiltrative abnormalities |

**Table S2.** Trimming and polishing parameters

| **Mapping to reference parameters** | **Values** |
| --- | --- |
| Guppy (MinKNOW interface) | Standard parameters and selection of discard middle and require two barcodes |
| Porechop | porechop -i input_reads.fastq -b output_dir --discard_middle --require_two_barcodes |
| Filtlong | filtlong --min_length 300 --keep_percent 90 input.fastq > output.fastq |
| Minimap 2 | minimap2 -ax map-ont |
| Racon | racon input.fastq overlaps.sam target_sequences.fasta --no-trimming -m 8 -x -6 -g -8 -w 500 |
| Medaka (default settings) | medaka_consensus -i reads.fastq -d draft_consensus.fasta -o outdir -t 4 -m r941_min_high_g360 |

**Table S3.** Mapping to the EV-C104 reference using CLC Genomics Workbench v20.0.3

| **Mapping to reference parameters** | **Values** |
| --- | --- |
| Masking mode | No masking |
| Match score | 1 |
| Mismatch cost | 2 |
| Cost of insertions and deletions | Linear gap cost |
| Insertion cost | 3 |
| Deletion cost | 3 |
| Length fraction | 0.7 |
| Similarity fraction | 0.8 |
| Global alignment | No |
| Non-specific match handling | Map randomly |
| Output mode | Create stand-alone read mappings |
| Create report | Yes |
| Collect unmapped reads | No |

**Table S4.** Creating a maximum likelihood tree using CLC Genomics Workbench v20.0.3

| **Maximum likelihood tree parameters** | **Values** |
| --- | --- |
| Construction method | Neighbor Joining |
| Existing start tree | Not set |
| Nucleotide substitution model | General Time Reversible |
| Protein substitution model | WAG |
| Transition / transversion ratio | 2 |
| Include rate variation | Yes |
| Number of substitution rate categories | 4 |
| Gamma distribution parameter | 1 |
| Estimate substitution rate parameter(s) | Yes |
| Estimate topology | Yes |
| Estimate gamma distribution parameter | Yes |
| Perform bootstrap analysis | Yes |
| Replicates | 1,000 |

**Table S5.** Creating primer binding sites and fragments using CLC Genomics Workbench v20.0.3

| **Primer binding sites and fragments parameters** | **Values** |
| --- | --- |
| Number of consecutive base pairs required in 3’ end | 8 |
| Minimum number of base pairs required for a match | 10 |
| Exact match | No |
| Binding sites annotations | Yes |
| Create binding sites table | Yes |
| Create fragment table | Yes |
| Min. fragment length | 100 |
| Max. fragment length | 4000 |
| Concentration of monovalent cations in buffer | 100 |
| Concentration of primer or probe | 200 |

**Table S6.** Performing a Fixed Ploidy Variant Detection using CLC Genomics Workbench v20.0.3

| **Fixed Ploidy Variant Detection parameter** | **Values** |
| --- | --- |
| Ploidy | 1 |
| Required variant probability (%) | 80 |
| Ignore positions with coverage above | 100000 |
| Restrict calling to target regions | false |
| Ignore broken pairs | true |
| Ignore non-specific matches | Reads |
| Minimum read length | 20 |
| Minimum coverage | 100 |
| Minimum count | 75 |
| Minimum frequency (%) | 75 |
| Base quality filter | false |
| Neighborhood radius | 5 |
| Minimum central quality | 20 |
| Minimum neighborhood quality | 15 |
| Read direction filter | false |
| Direction frequency (%) | 5 |
| Relative read direction filter | false |
| Significance (%) | 1 |
| Read position filter | false |
| Significance (%) | 1 |
| Remove pyro-error variants | false |
| In homopolymer regions with minimum length | 3 |
| With frequency below | 0.8 |

**Table S7**. Overview of primer binding analysis in Respiratory samples 1-4

| **Assay** | **Forward primer (5’end)** | **Reverse primer (3’end)** | **Fragment length (nt)** | **Forward primer  mismatches** | **Reverse primer  mismatches** |
| --- | --- | --- | --- | --- | --- |
| **PCR primers** | Ent224:GCIATGYTIGGIACICAYRT | Ent222:CICCIGGIGGIAYRWACAT | 920 | 0 | 3 |
| **Nested PCR primers** | AN89:CCAGCACTGACAGCAGYNGARAYNGG | AN88:TACTGGACCACCTGGNGGNAYRWACAT | 378 | 2 | 7 |
| **Sequencing primers** | AN233:TACTGGACCACCTGG | AN232:CCAGCACTGACAGCA | 378 | 2 | 7 |

**Table S8**. Detected intra-host variants between sample dates.

| **Sample ID** | **Region** | **CDS gene** | **Protein Function** | **Change in CDS** | **Non-synonymous** | **Amino acid change** | **Coding region change** | **Frequency (%)** |
| --- | --- | --- | --- | --- | --- | --- | --- | --- |
| R2 | 670 | 5'UTR | Regulation of translation |  | - |  | -33T>C | 83.91 |
|  | 1728 | VP3 | Forms an icosahedral capsid |  | No |  | 1026G>A | 82.76 |
|  | 2863 | VP1 | Interaction and attachment with host cell receptor | Frameshift | Yes | Asp723 | 2167delG | 75.69 |
|  | 4392 | 2C | Structural rearrangements of intracellular membranes. |  | No |  | 3690T>C | 76.70 |
|  | 5229 | 3A | Involved in viral replication |  | No |  | 4527C>T | 87.16 |
|  | 6204 | 3D | Involved in viral replication and maturation. |  | Yes | Asp1834Glu | 5502T>A | 93.75 |
| R3 | 670 | 5'UTR | Regulation of translation |  | - |  | -33T>C | 94.09 |
|  | 1599 | VP2 | Forms an icosahedral capsid |  | No |  | 897C>T | 84.35 |
|  | 1728 | VP3 | Forms an icosahedral capsid |  | No |  | 1026G>A | 87.33 |
|  | 1962 | VP3 | Forms an icosahedral capsid |  | No |  | 1260A>G | 90.13 |
|  | 2232 | VP3 | Forms an icosahedral capsid |  | No |  | 1530A>G | 92.44 |
|  | 2692 | VP1 | Interaction and attachment with host cell receptor |  | Yes | Val664Ile | 1990G>A | 93.03 |
|  | 2863 | VP1 | Interaction and attachment with host cell receptor | Frameshift | Yes | Asp723 | 2167delG | 77.86 |
|  | 3456 | 2A | Cysteine protease |  | No |  | 2754C>T | 94.13 |
|  | 4350 | 2C | Structural rearrangements of intracellular membranes. |  | No |  | 3648C>T | 83.59 |
|  | 4392 | 2C | Structural rearrangements of intracellular membranes. |  | No |  | 3690T>C | 94.03 |
|  | 5229 | 3A | Involved in viral replication |  | No |  | 4527C>T | 90.71 |
|  | 6042 | 3D | Involved in viral replication and maturation. |  | No |  | 5340G>A | 77.51 |
|  | 6204 | 3D | Involved in viral replication and maturation. |  | Yes | Asp1834Glu | 5502T>A | 94.20 |
|  | 7209 | 3D | Involved in viral replication and maturation. |  | No |  | 6507C>T | 92.06 |
| R4 | 670 | 5'UTR | Regulation of translation |  | - |  | -33T>C | 91.89 |
|  | 846 | VP4 | Forms an icosahedral capsid |  | No |  | 144A>G | 86.43 |
|  | 1038 | VP2 | Forms an icosahedral capsid |  | No |  | 336T>C | 84.11 |
|  | 1326 | VP2 | Forms an icosahedral capsid |  | No |  | 624T>C | 93.50 |
|  | 1599 | VP2 | Forms an icosahedral capsid |  | No |  | 897C>T | 90.18 |
|  | 1728 | VP3 | Forms an icosahedral capsid |  | No |  | 1026G>A | 86.16 |
|  | 1962 | VP3 | Forms an icosahedral capsid |  | No |  | 1260A>G | 78.36 |
|  | 2232 | VP3 | Forms an icosahedral capsid |  | No |  | 1530A>G | 88.66 |
|  | 2692 | VP1 | Interaction and attachment with host cell receptor |  | Yes | Val664Ile | 1990G>A | 92.07 |
|  | 2863 | VP1 | Interaction and attachment with host cell receptor | Frameshift | Yes | Asp723 | 2167delG | 78.64 |
|  | 3456 | 2A | Cysteine protease |  | No |  | 2754C>T | 89.40 |
|  | 3762 | 2A | Cysteine protease |  | No |  | 3060G>A | 87.82 |
|  | 4245 | 2C | Structural rearrangements of intracellular membranes. |  | No |  | 3543T>C | 90.72 |
|  | 4350 | 2C | Structural rearrangements of intracellular membranes. |  | No |  | 3648C>T | 91.66 |
|  | 4392 | 2C | Structural rearrangements of intracellular membranes. |  | No |  | 3690T>C | 91.38 |
|  | 4754 | 2C | Structural rearrangements of intracellular membranes. | Frameshift | Yes | Pro1353 | 4058delC | 75.64 |
|  | 4842 | 2C | Structural rearrangements of intracellular membranes. |  | No |  | 4140G>A | 79.97 |
|  | 5175 | 3A | Involved in viral replication |  | No |  | 4473T>C | 85.72 |
|  | 5229 | 3A | Involved in viral replication |  | No |  | 4527C>T | 87.21 |
|  | 6126 | 3D | Involved in viral replication and maturation. |  | No |  | 5424T>C | 92.35 |
|  | 6204 | 3D | Involved in viral replication and maturation. |  | Yes | Asp1834Glu | 5502T>A | 93.02 |
|  | 7134 | 3D | Involved in viral replication and maturation. |  | Yes | Glu2144Asp | 6432G>C | 94.06 |

**Abbreviations:** Respiratory 2; R2, Respiratory 3; R3, Respiratory 4; R4. Fixed Ploidy algorithm with 80% variant probability and 75% minimum frequency on CLC Genomics Workbench.


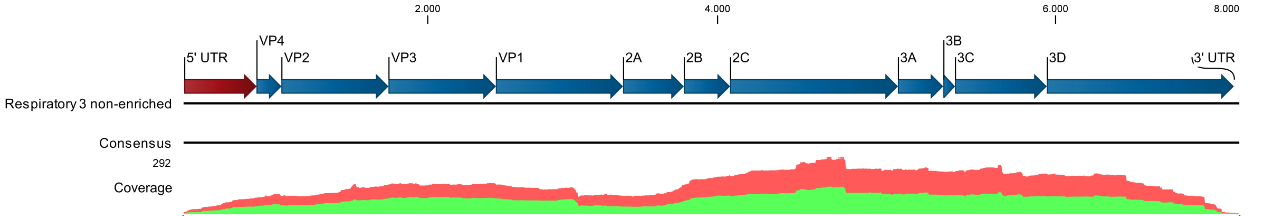


**Figure S1.** EV-C104 genome coverage of Respiratory 3 (non-enriched) sample**.** Read coverage obtained from read mappings. Red, single read in reverse direction; Green, single read in forward direction. Open reading frame is depicted with blue arrows. Untranslated regions are depicted with red arrows.


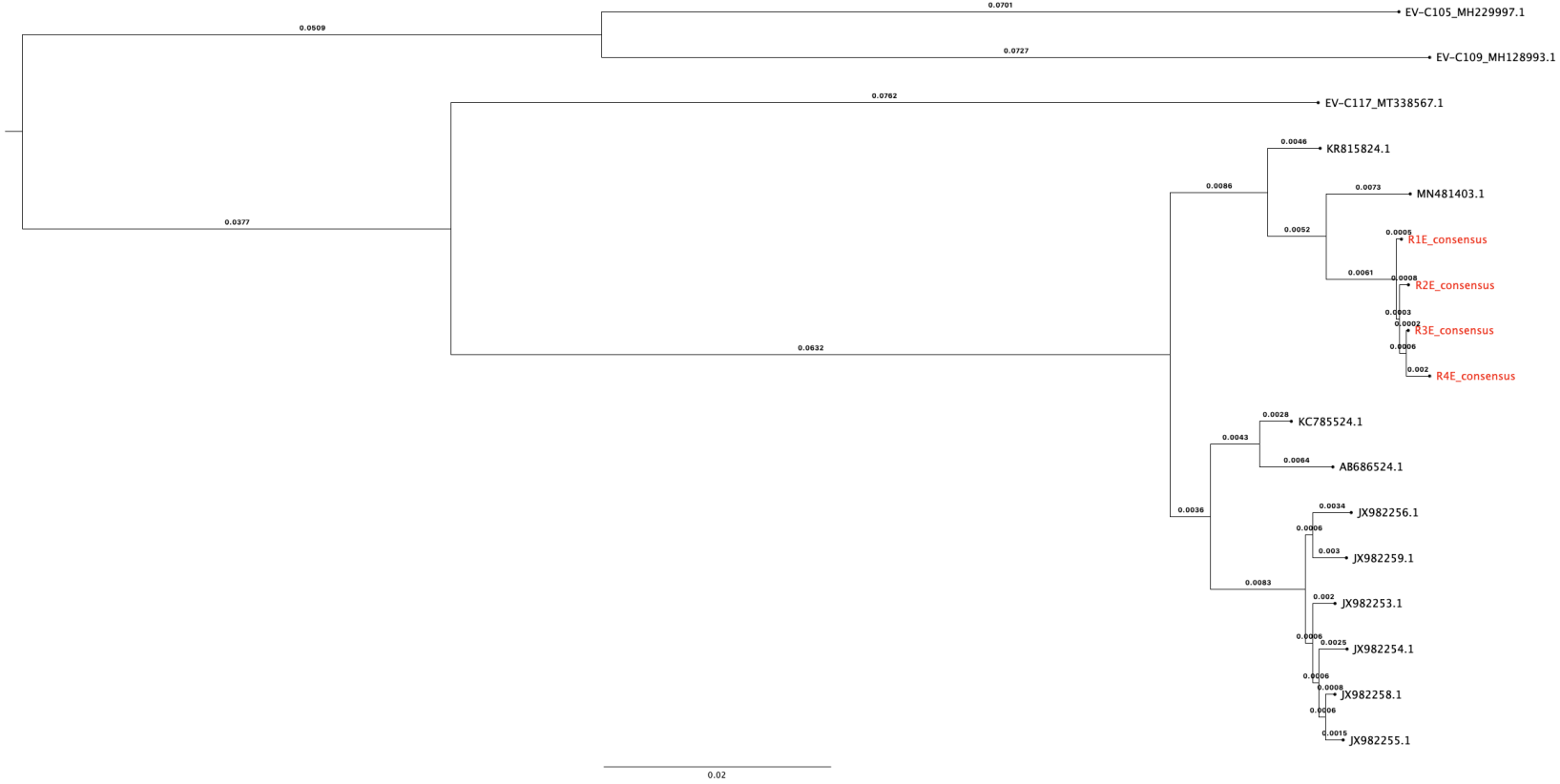
**Figure S2**. Phylogenetic reconstruction with substitution rates. Maximum-likelihood phylogenetic tree inferred from EV-C104 complete/near-complete sequences. Patient samples (in red with the letter E to indicate they have been enriched), ten complete EV-C104 genomes and lastly, three EV-C genomes from GenBank served as outgroups. CLC was used to generate the alignment and construct the tree. A General Time Reversible substitution model and a gamma distribution with invariant sites and 1000 bootstraps were used.


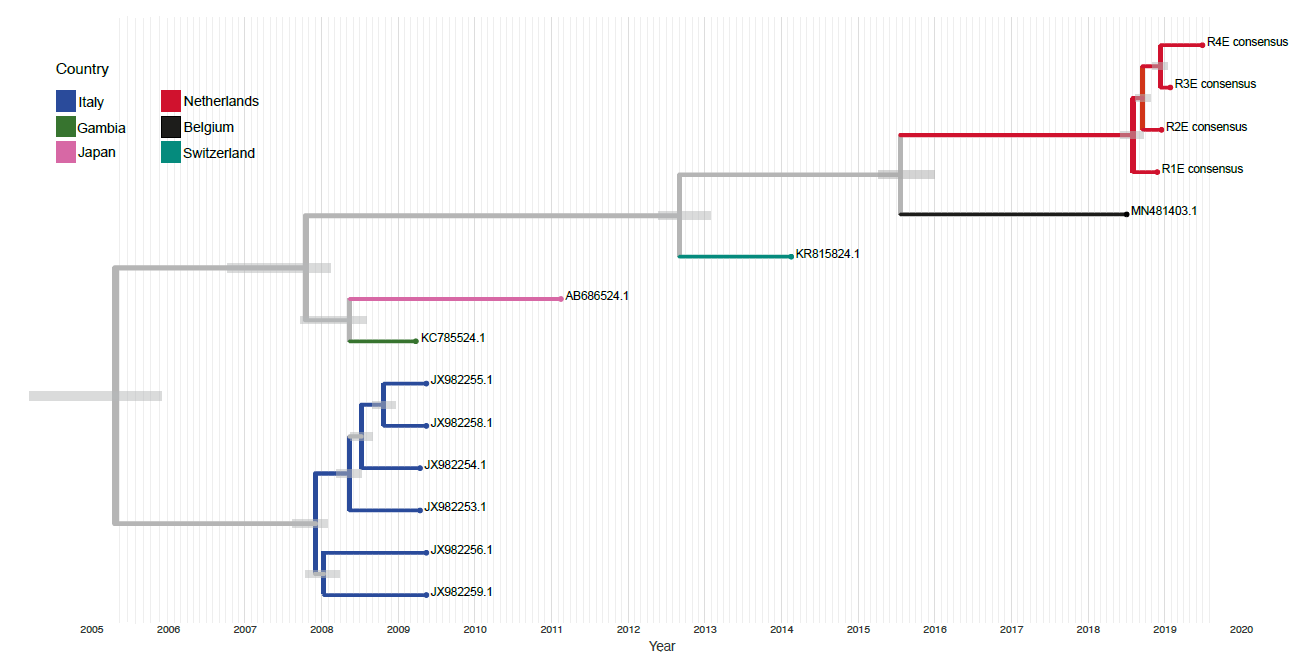


**Figure S3**. Time scaled phylogeny of EV-C104 near-full genomes. The augur pipeline implemented in Nextstrain was run using the aligner MAFFT and the phylodynamic package TreeTime, followed by visualization using auspice. A coalescent skyline model with a strict molecular clock and best root fitted was performed. Enterovirus sequences are highlighted according to country: Sequences from the Netherlands (this study) are red, sequences from Belgium are black, sequences from Switzerland are turquoise, sequences from Japan are pink, sequences from Gambia are green and sequences from Italy are blue. The grey boxes at each node indicate the confidence intervals for the estimated TMRCA.
